# SpoIIQ-dependent localization of SpoIIE contributes to septal stability and compartmentalization during the engulfment stage of *Bacillus subtilis* sporulation

**DOI:** 10.1128/jb.00220-24

**Published:** 2024-06-21

**Authors:** Behzad Dehghani, Christopher D. A. Rodrigues

**Affiliations:** 1 School of Life Sciences, University of Warwick, Coventry, United Kingdom; Geisel School of Medicine at Dartmouth, Hanover, New Hampshire, USA

**Keywords:** sporulation, engulfment, peptidoglycan, peptidoglycan remodeling, compartmentalization

## Abstract

During spore development in bacteria, a polar septum separates two transcriptionally distinct cellular compartments, the mother cell and the forespore. The conserved serine phosphatase SpoIIE is known for its critical role in the formation of this septum and activation of compartment-specific transcription in the forespore. Signaling between the mother cell and forespore then leads to activation of mother cell transcription and a phagocytic-like process called engulfment, which involves dramatic remodeling of the septum and requires a balance between peptidoglycan synthesis and hydrolysis to ensure septal stability and compartmentalization. Using *Bacillus subtilis*, we identify an additional role for SpoIIE in maintaining septal stability and compartmentalization at the onset of engulfment. This role for SpoIIE is mediated by SpoIIQ, which anchors SpoIIE in the engulfing membrane. A SpoIIQ mutant (SpoIIQ Y28A) that fails to anchor SpoIIE, results in septal instability and miscompartmentalization during septal peptidoglycan hydrolysis, when other septal stabilization factors are absent. Our data support a model whereby SpoIIE and its interactions with the peptidoglycan synthetic machinery contribute to the stabilization of the asymmetric septum early in engulfment, thereby ensuring compartmentalization during spore development.

**IMPORTANCE:**

Bacterial sporulation is a complex process involving a vast array of proteins. Some of these proteins are absolutely critical and regulate key points in the developmental process. Once such protein is SpoIIE, known for its role in the formation of the polar septum, a hallmark of the early stages of sporulation, and activation of the first sporulation-specific sigma factor, σF, in the developing spore. Interestingly, SpoIIE has been shown to interact with SpoIIQ, an important σF-regulated protein that functions during the engulfment stage. However, the significance of this interaction has remained unclear. Here, we unveil the importance of the SpoIIQ-SpoIIE interaction and identify a role for SpoIIE in the stabilization of the polar septum and maintenance of compartmentalization at the onset of engulfment. In this way, we demonstrate that key sporulation proteins, like SpoIIQ and SpoIIE, function in multiple processes during spore development.

## INTRODUCTION

In response to nutrient scarcity, some bacteria belonging to the phylum Firmicutes initiate a process called sporulation. This 7–8-hour process begins with polar division which divides the starving cell into transcriptionally distinct cellular compartments, a larger mother and a smaller forespore. Next, the forespore is internalized inside the mother cell in a highly complex, phagocytic-like process called engulfment, forming a cell-within-a-cell. Within the mother cell, the forespore matures by the addition of thick, protective envelope layers. Upon maturation, the mother cell lyses, releasing the dormant spore into the environment, where it remains dormant until nutrient-sensing signals germination, followed by the outgrowth of the bacterium to vegetative growth. In this work, we focus on early events of the sporulation process that ensure the stability of the polar septum at the onset of engulfment.

Sporulation is initiated by a key regulator, Spo0A (1, 2). Spo0A–P directly controls the expression of approximately 120 genes (according to ChlP data), but indirectly controls the expression of many more genes, including some encoding the sporulation sigma factors σF, σE, σG, and σK, which play a major role in activating compartment-specific transcriptional programs. The early stages of development are governed by σF and σE, in the forespore and mother cell, respectively (3). At later stages of development, when the engulfment of the forespore is completed, σG and σK are then activated in the forespore and mother cell, respectively (3). Among these sigma factors, σF is particularly important as it commits developing forespores to the sporulation pathway (4).

**Fig 1:**
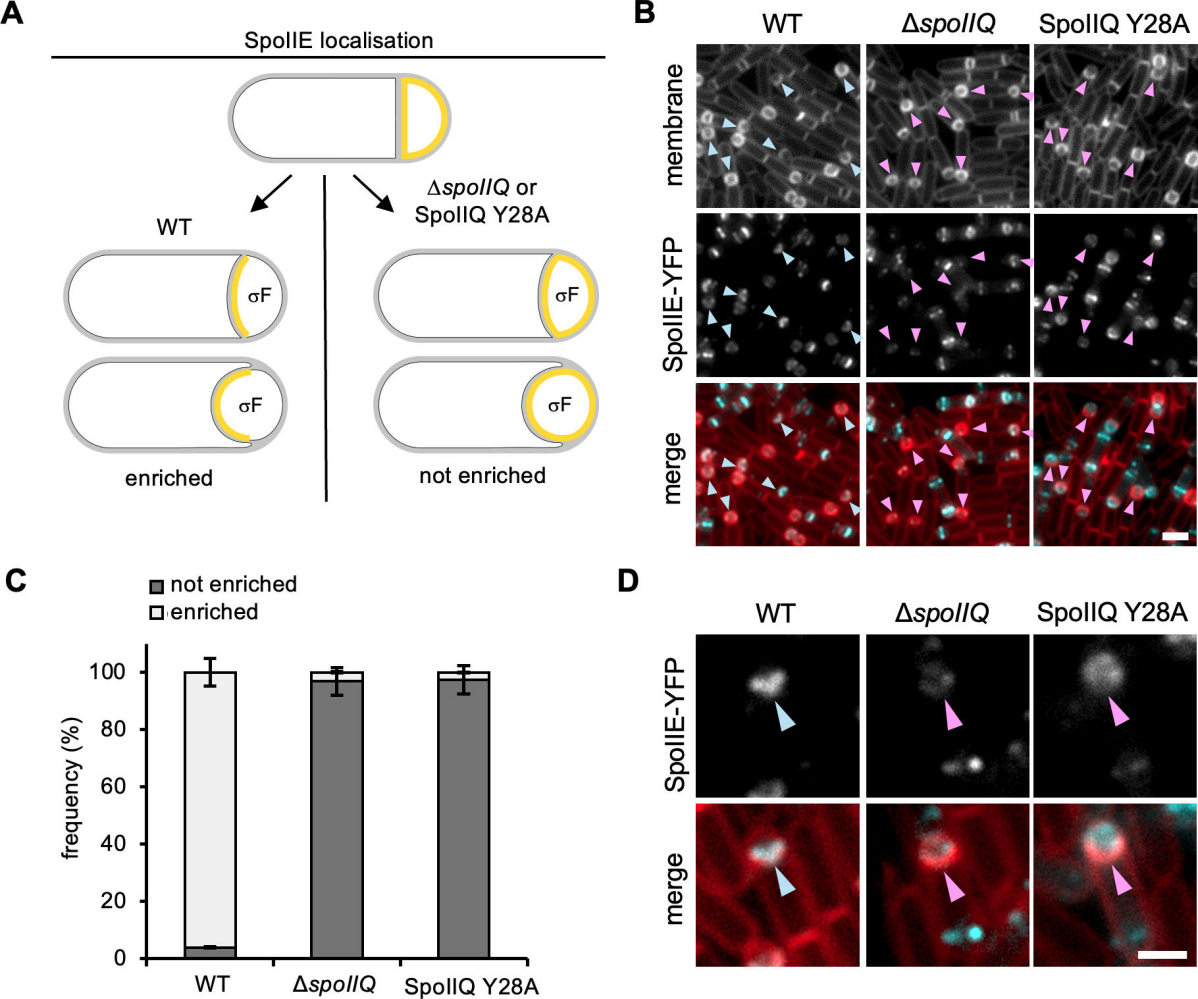
SpoIIQ and SpoIIQ Y28 are required for SpoIIE localization at the engulfing membrane. (**A**) Schematic representation of the localization of SpoIIE. Upon completion of asymmetric division, SpoIIE is localized in all forespore membranes. Upon σF activation, and subsequent production of SpoIIQ, in WT (wild-type) cells, SpoIIE becomes enriched in the engulfing membrane. In cells lacking SpoIIQ, or cells harboring the SpoIIQ Y28A mutation, SpoIIE fails to become enriched in the engulfing membrane. (**B**) Representative images of SpoIIE-YFP localization in WT, the Δ*spoIIQ* mutant, and the SpoIIQ Y28A mutant. Blue arrowheads point to sporangia where SpoIIE-YFP is enriched in the engulfing membrane. Pink arrowheads point to sporangia where SpoIIE-YFP exhibits no enrichment in the engulfing membrane. SpoIIE-YFP is pseudocolored in cyan. Scale bar, 2 µm. (**C**) Histogram showing the average frequency of cells exhibiting enrichment and no enrichment of SpoIIE-YFP at the engulfing membrane, in WT, the Δ*spoIIQ* mutant, and the SpoIIQ Y28A mutant at T2 [±standard deviation (SD) of three biological replicates, >100 cells per replicate]. (**D**) Representative zoomed-in examples of SpoIIE-YFP localization in WT, the Δ*spoIIQ* mutant, and the SpoIIQ Y28A mutant. SpoIIE-YFP is pseudocolored in cyan. Scale bar, 1 µm. For more examples, refer to Fig. S1 and S2.

σF activation requires the SpoIIE protein, which has at least two known functions (5–7). First, it is required for the formation of the polar septum and, second, it plays a vital role in the activation of σF. This large protein (92 kDa) contains three distinct domains, the membrane-spanning domain (I), the central domain (II), and the conserved PP2C-like phosphatase domain (III) (5–7). It has been shown that the positioning of FtsA and FtsZ protein filaments required for the initiation of polar cell division is regulated by SpoIIE’s colocalization with FtsZ (8). Following polar division, SpoIIE is temporarily freed from the septum and localizes to all membranes within the forespore compartment (9). Upon σF activation and initiation of engulfment, SpoIIE then relocalizes to the engulfing septal membranes (9). It has been shown that SpoIIQ, produced in the forespore under σF-control, interacts with SpoIIE and is required for its relocalization to the engulfing septal membrane (9). Indeed, in sporulating cells lacking SpoIIQ or cells harboring a SpoIIQ mutant with an amino acid substitution in the transmembrane domain of SpoIIQ (SpoIIQ Y28A), SpoIIE fails to localize to the engulfing septal membrane (Fig. 1A) (9, 10). Intriguingly, the significance of the interaction between SpoIIQ and SpoIIE, and SpoIIE’s localization in the engulfing membrane, remains mysterious. Current data suggest that the SpoIIQ Y28A mutant exhibits a modest decrease in SpoIIE-GFP protein levels (by immunoblotting), a slight decrease in σG activity, and almost wild-type levels of heat-resistant spores (10). Since σG activity occurs after engulfment completion, the SpoIIQ-dependent relocalization of SpoIIE to the septal membranes at the onset of engulfment suggests that SpoIIE plays a role during this important stage of spore development.

Multiple studies have shown that engulfment requires multiple processes to occur efficiently (11–14). These include peptidoglycan (henceforth PG) hydrolysis and synthesis, membrane synthesis, and a biophysical ratchet established by the intercellular interaction between SpoIIQ in the forespore and SpoIIIAH in the mother cell (known as the SpoIIIAH-SpoIIQ ratchet) (11, 14). PG hydrolysis occurs principally through the activity of the mother cell-produced DMP complex (named after the proteins in this complex), composed of SpoIID, SpoIIM, and SpoIIP, which localizes at the polar septum and thins septal PG (15, 16). While the exact players involved in PG synthesis during engulfment remain less well defined, multiple studies suggest PG synthesis is important for the process to occur efficiently (12, 14, 17). The SpoIIIAH-SpoIIQ ratchet, on the other hand, bridges the mother cell and forespore membrane and facilitates efficient migration of the mother cell membrane around the forespore (13, 18).

Importantly, during the early stages of engulfment, and as the PG within the polar septum becomes remodeled, a copy of the chromosome is translocated across a pore in the septum and into the forespore by the DNA translocase SpoIIIE (19). We recently found that PG synthesis and hydrolysis at the onset of engulfment must be balanced to ensure stabilization of this septal pore and cytoplasmic compartmentalization of the forespore (20). It was shown that septal stabilization and compartmentalization at the onset of engulfment are mediated by SpoIIIE and two proteins it interacts with are SpoIIIM and PbpG (20). SpoIIIM is a mother cell-produced LysM domain-containing protein that likely binds PG, and PbpG is a Class A penicillin-binding protein produced in the forespore (20). Together SpoIIIM and PbpG maintain the size of the septal pore, and therefore, forespore compartmentalization, by counteracting the hydrolytic activity of the DMP complex on PG surrounding the septal pore (20). In the absence of SpoIIIE, SpoIIIM, and PbpG, the septal pore enlarges due to the activity of the DMP complex and results in leakage of the forespore cytoplasm into the mother cell (known as miscompartmentalization) and failure to retain the chromosome within the forespore.

Interestingly, a more severe defect called septal retraction was observed in the absence of SpoIIIE, SpoIIIM, and PbpG, if SpoIIQ was also absent (20). Septal retraction occurs following the formation of the polar septum and σF activation in the forespore and is also dependent on the DMP complex (20). Septal retraction results in sporangia that have activated σF (i.e., are miscompartmentalized) but exhibit no distinct forespore compartment; instead, in some cells there are vestigial septa (20). It was hypothesized that septal retraction occurs due to the simultaneous loss of two septal stabilization mechanisms at the onset of DMP complex activity: (i) the coordination between chromosome translocation and PG synthesis mediated by SpoIIIE, SpoIIIM, and PbpG which reinforces the septum through protein-PG interactions and PG synthesis and (ii) the SpoIIIAH-SpoIIQ ratchet (20). However, as mentioned above, lack of SpoIIQ also causes SpoIIE mislocalization, raising the possibility that SpoIIQ-dependent relocalization of SpoIIE to the engulfing septal membranes contributes to the stabilization of the septum at the onset of engulfment, thus preventing septal retraction.

In this work, taking advantage of the SpoIIQ Y28A mutant, we show that SpoIIE localization to the engulfing membrane is required to prevent septal retraction and miscompartmentalization, when other septal stabilization mechanisms are absent. Our data reveal an additional role for SpoIIE during sporulation; we propose a model whereby SpoIIE interactions with the PG synthetic machinery contribute to septal stability upon the initiation of engulfment (Fig. 7). Collectively our data suggest that there are two pathways involving PG synthesis that contribute to septal stabilization and compartmentalization at the onset of engulfment, the SpoIIIE pathway and the SpoIIQ pathway that involves SpoIIE (Fig. 7).

## RESULTS

### SpoIIQ Y28 is required for the localization of SpoIIE in the engulfing membrane

Previous worked had shown that SpoIIQ and specifically SpoIIQ Y28 are required for the localization of the SpoIIE to the engulfing membrane (9, 10). Furthermore, SpoIIQ Y28A results in a modest decrease in SpoIIE-GFP protein levels at early stages of development (assessed by immunoblot) (10). However, it remains unclear if the absence of SpoIIQ and the SpoIIQ Y28A mutant results in mislocalization of SpoIIE in all cells and if the mislocalization defect of each mutant is the same. To this end, we examined a previously characterized SpoIIE-YFP fluorescent fusion (9) (used as the sole source of SpoIIE), in an otherwise WT background, in the Δ*spoIIQ* mutant and in a strain where SpoIIQ Y28A is expressed as the sole source of SpoIIQ from an ectopic locus (henceforth SpoIIQ Y28A). We examined cells 2 hours after the onset of sporulation (T2), since at this time point many sporangia have begun engulfment (illustrated by a curved septal membrane) and WT cells would be expected to exhibit localization of SpoIIE-YFP in the engulfing membrane. Consistent with previous data, SpoIIE-YFP was enriched in the engulfing membrane of WT cells in 96% of the sporangia (Fig. 1B through D). In the Δ*spoIIQ* mutant and in the SpoIIQ Y28A background, SpoIIE-YFP had no enrichment in the engulfing membrane in most cells (97% in both mutants) (Fig. 1B through D; Fig. S1).

While the absence of SpoIIQ and SpoIIQ Y28A resulted in no enrichment of SpoIIE-YFP in the engulfing membrane, we noticed differences in how SpoIIE-YFP was localized in the forespore membranes. Quantification of SpoIIE-YFP signal intensity across the forespore membranes in WT cells showed enrichment of SpoIIE-YFP signal that coincides with the engulfing membranes (Fig. S1A and B). However, in the SpoIIQ mutant background, there was no such enrichment; in some instances, however, we observed faint localization of SpoIIE-YFP at the leading edge, or ahead of the engulfing membrane (Fig. S1A). Interestingly, in the SpoIIQ Y28A mutant, localization of SpoIIE-YFP at the leading edge or ahead of the engulfing membrane appeared more pronounced (Fig. S1A and B). These localization defects were confirmed at higher resolution using Structured illumination microscopy (SIM) (Fig. S2). Furthermore, and consistent with previous data showing that SpoIIQ is required for SpoIIE stability (10), the absence of SpoIIQ resulted in a decrease of SpoIIE-YFP signal intensity (Fig. S1C). This was not the case with SpoIIQ Y28A which retained near WT levels of SpoIIE-YFP signal (Fig. S1C).

### SpoIIQ Y28A enhances septal retraction in the absence of SpoIIIE

**Fig 2:**
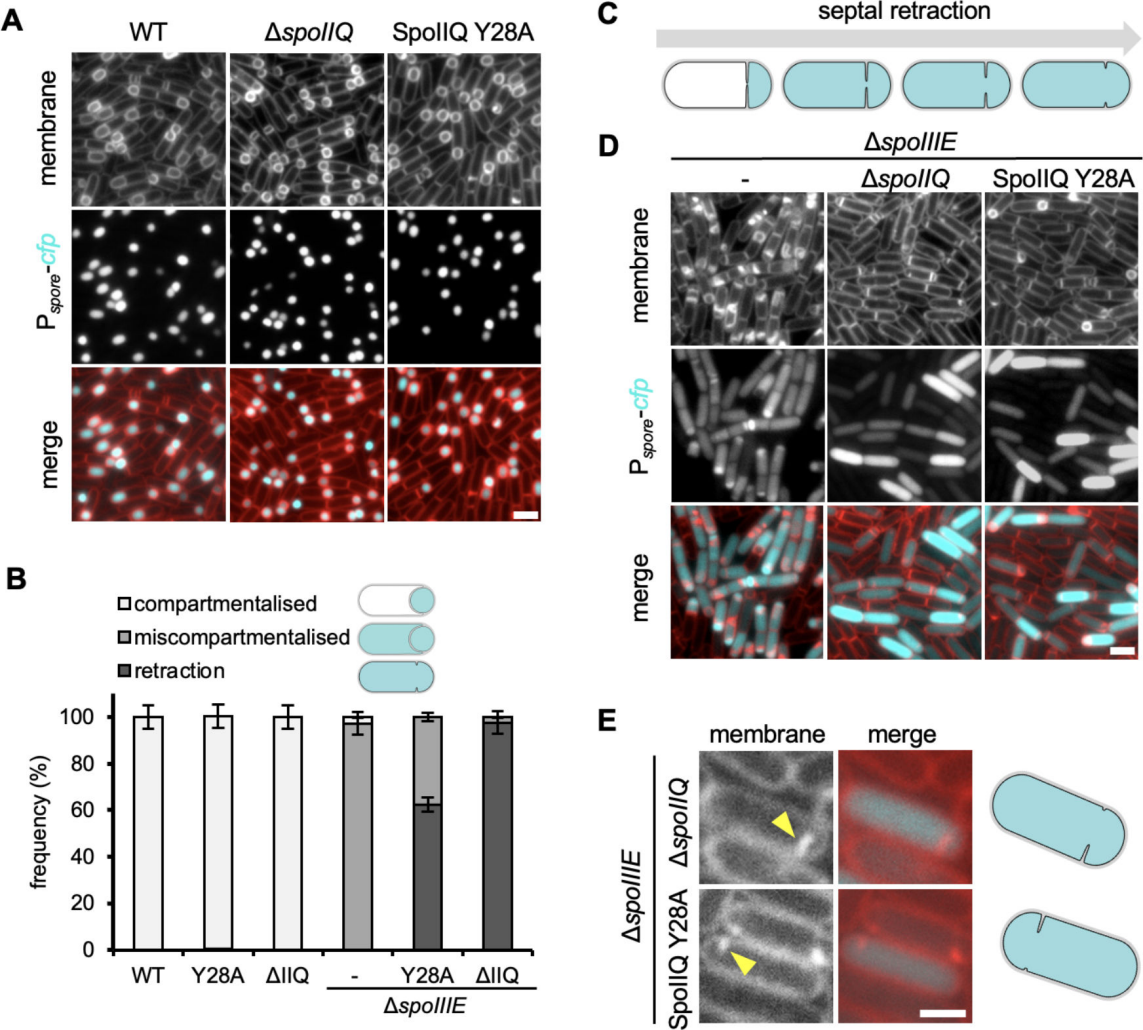
Septal retraction occurs in the SpoIIQ Y28A mutant. (**A**) Representative images of compartmentalization in WT, Δ*spoIIQ*, and SpoIIQ Y28A mutant. Scale bar is 2 µm. (**B**) Histogram showing the average frequency of compartmentalization, miscompartmentalization, and septal retraction at T3 in Δ*spoIIIE*, Δ*spoIIIE* Δ*spoIIQ*, and Δ*spoIIIE* SpoIIQ Y28A (±SD of three biological replicates, >100 cells per replicate). (**C**) Schematic representation of septal retraction, illustrating that as septal retraction progresses, CFP fluorescence (cyan) leaks from the forespore to fill the entire sporangium. (**D**) Representative images of miscompartmentalization and septal retraction in Δ*spoIIIE*, Δ*spoIIIE* Δ*spoIIQ*, and Δ*spoIIIE* SpoIIQ Y28A. Scale bar is 2 µm. (**E**) Representative zoomed-in examples of septal retraction in Δ*spoIIIE* Δ*spoIIQ* and Δ*spoIIIE* SpoIIQ Y28A at T3. Yellow arrowheads point to retracted septa. Scale bar, 1 µm. Schematic representations of cells are shown on the right.

Previously, we showed that the absence of both *spoIIIE* and *spoIIQ* causes a dramatic phenotype called septal retraction, where shortly after activation of σF and the initiation of engulfment, the septal membranes retract (20) (Fig. 2C). This results in sporangia that have activated σF but with no distinct forespores (20). To test the possibility that septal retraction arises as a result of SpoIIE mislocalization, we compared septal retraction in the Δ*spoIIIE* Δ*spoIIQ* double mutant, to the Δ*spoIIIE* SpoIIQ Y28A double mutant. We used a strain background harboring a fluorescent reporter in the forespore (P*_spoIIQ_-cfp*) and monitored spore development 3 hours after the onset of starvation (T3), when septal retraction in the Δ*spoIIIE* Δ*spoIIQ* double mutant is observed in almost all sporangia containing CFP (cyan fluorescent protein) signal (20).

Like Δ*spoIIQ*, the SpoIIQ Y28A mutant did not impact compartmentalization (Fig. 2A). Consistent with previous results (20), Δ*spoIIIE* resulted in sporangia with miscompartmentalized CFP signal and deformed forespores, while Δ*spoIIQ* Δ*spoIIIE* resulted in sporangia exhibiting septal retraction (miscompartmentalization with no visible forespores) (Fig. 2D). In the Δ*spoIIIE* strain harboring SpoIIQ Y28A, we observed a large proportion of sporangia exhibiting septal retraction (Fig. 2D) and quantification showed that in Δ*spoIIIE* SpoIIQ Y28A double mutant, 62% of sporangia exhibited septal retraction, while the remainder (38%) were miscompartmentalized (Fig. 2B). For comparison, in the Δ*spoIIIE* Δ*spoIIQ* double mutant almost all sporangia exhibited septal retraction (98%, Fig. 2B). Furthermore, as observed previously for the Δ*spoIIQ* Δ*spoIIIE* double mutant (20), we also observed vestiges of septa in the Δ*spoIIIE* SpoIIQ Y28A double mutant (Fig. 2E). This result suggests that localization of SpoIIE to the engulfing membrane by SpoIIQ plays an important role in septal stabilization.

### SpoIIQ Y28A increases septal retraction in a SpoIIIE hypomorph

The above data suggest a role for SpoIIQ-mediated SpoIIE localization in septal stability and prevention of septal retraction. However, since cells lacking SpoIIIE exhibit morphological defects and are all miscompartmentalized, we wanted to test if SpoIIQ-mediated localization of SpoIIE to the engulfing membrane also plays a role in compartmentalization and septal stabilization in a mutant background that exhibits a less pronounced defect. To test this, we took advantage of a SpoIIIE hypomorphic allele (SpoIIIE D584A) which results in miscompartmentalization in approximately 20% of the cells (20). We compared compartmentalization (and septal retraction) in the SpoIIIE D584A cells, that also lacked SpoIIQ or contained the SpoIIQ Y28A allele at T3.

**Fig 3:**
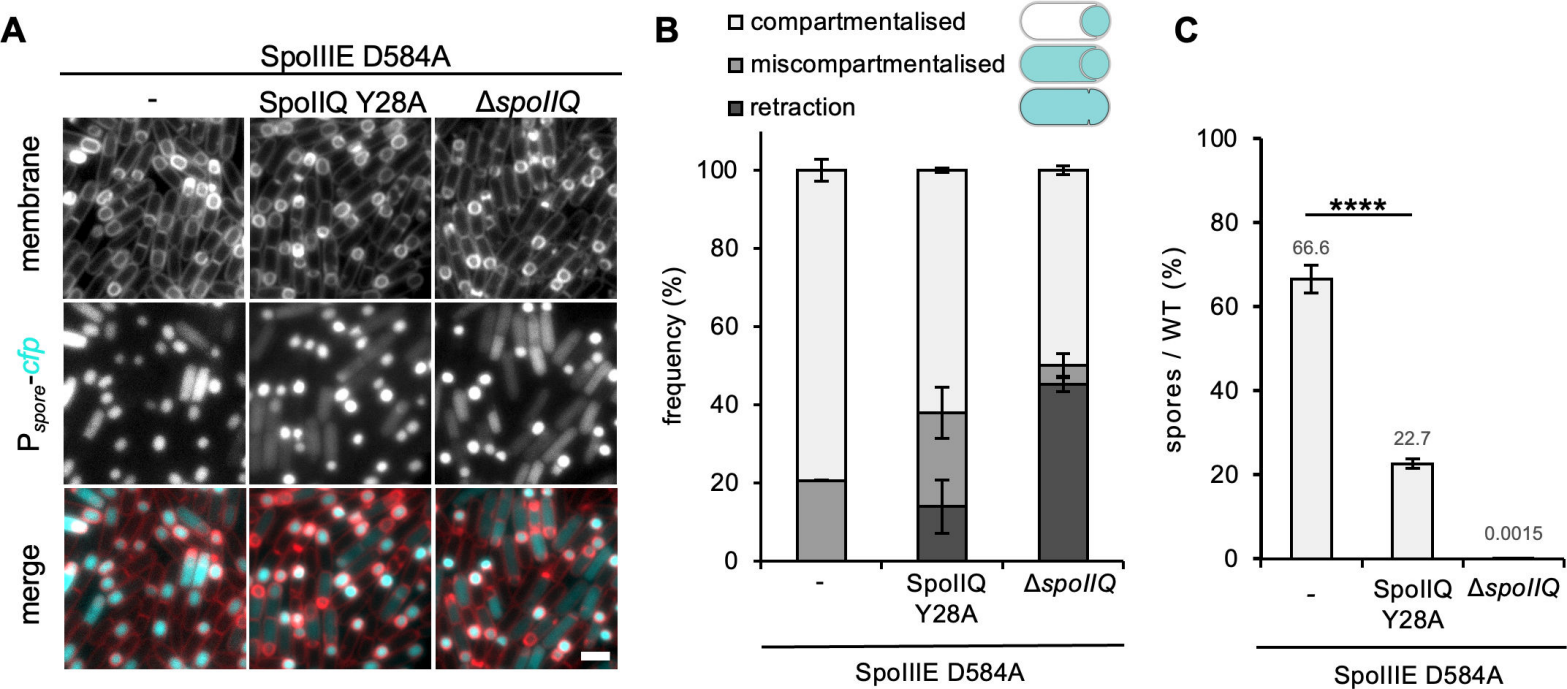
The SpoIIQ Y28A mutant enhances septal instability in a SpoIIIE hypomorph. (**A**) Representative images of the SpoIIIE D584A mutant, the SpoIIIE D584A mutant combined with the SpoIIQ Y28A mutant, or the Δ*spoIIQ* mutant. Scale bar is 2 µm. (**B**) Histogram showing the average frequency of compartmentalization, miscompartmentalization, and septal retraction at T3 in the SpoIIIE D584A mutant, the SpoIIIE D584A SpoIIQ Y28A double mutant, and the SpoIIIE D584A Δ*spoIIQ* double mutant (±SD of three biological replicates, >100 cells per replicate). (**C**) Average sporulation efficiency (% ±SD, *n* = 3) of the SpoIIIE D584A mutant, the SpoIIIE D584A SpoIIQ Y28A double mutant, and the SpoIIIE D584A Δ*spoIIQ* double mutant. Error bars represent the SD from three biological replicates. ****P < 0.0001 by Student’s *t*-test and one-way analysis of variance (ANOVA).

Consistent with previous results (20), SpoIIIE D584A resulted in 21% sporangia with miscompartmentalized CFP signal but with no visible septal retraction (Fig. 3A and B). Interestingly, combining SpoIIIE D584A with SpoIIQ Y28A resulted in a slight increase in miscompartmentalization (24%) but a noticeable increase in septal retraction (14%) (Fig. 3). In Δ*spoIIQ*, there was a higher frequency of septal retraction (45%) compared to SpoIIQ Y28A. Since the SpoIIQ Y28A mutant exacerbated the septal retraction defects of SpoIIIE D584A, we would expect to see a decrease in the formation of the heat-resistant spores compared to SpoIIIE D584A. Consistent with this prediction, combining SpoIIQ Y28A with SpoIIIE D584A resulted in a threefold reduction in sporulation efficiency compared to SpoIIIE D584A alone (Fig. 3C). These results suggest that SpoIIQ and SpoIIQ-mediated localization of SpoIIE to the engulfing membrane play an important role in the maintenance of compartmentalization and the formation of heat-resistant spores.

### SpoIIQ Y28A enhances septal retraction and miscompartmentalization in the absence of SpoIIIM and PbpG

**Fig 4:**
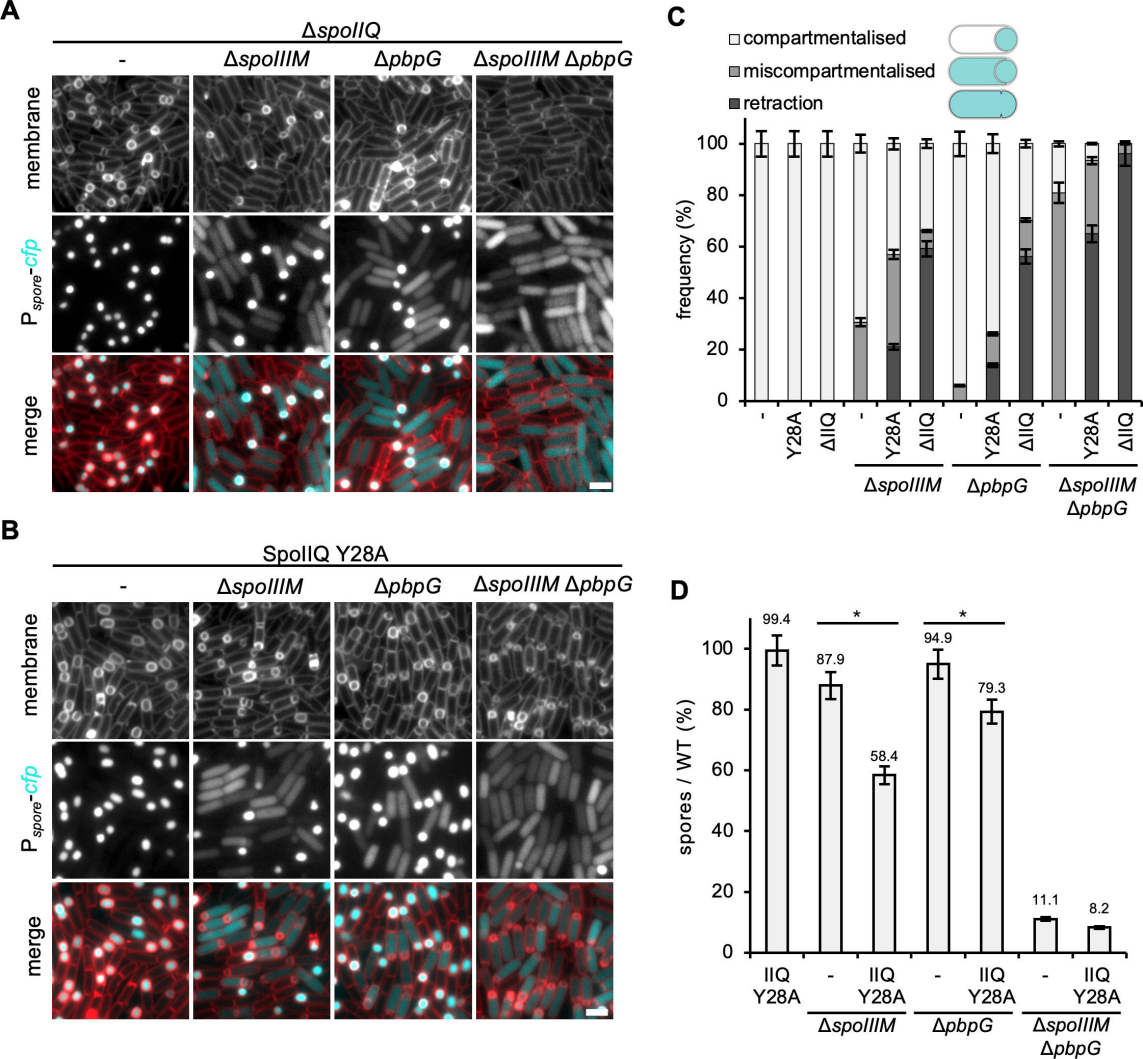
SpoIIQ Y28A enhances the septal stability and compartmentalization defects of SpoIIIM and PbpG mutants. (**A**) Representative images of septal retraction and miscompartmentalization in Δ*spoIIQ*, Δ*spoIIQ* Δ*spoIIIM*, Δ*spoIIQ* Δ*pbpG*, and Δ*spoIIQ* Δ*spoIIIM* Δ*pbpG*. Scale bar is 2 µm. (**B**) Representative septal retraction and miscompartmentalization in SpoIIQ Y28A, SpoIIQ Y28A Δ*spoIIIM*, SpoIIQ Y28A Δ*pbpG*, and SpoIIQ Y28A Δ*spoIIIM* Δ*pbpG*. Scale bar is 2 µm. (**C**) Histogram showing the average frequency (±SD of three biological replicates, >100 cells per replicate) of compartmentalization, miscompartmentalization, and septal retraction at T3 in mutants stated in (**A**) and (**B**). (**D**) Average sporulation efficiency (% ±SD, *n* = 3) of Δ*spoIIIM*, Δ*pbpG*, and Δ*spoIIIM* Δ*pbpG* mutants in an otherwise WT background (-) and SpoIIQ Y28A background (IIQ Y28A). Error bars represent the SD from three biological replicates. *P < 0.05 by Student’s *t*-test and one-way ANOVA.

Next, we sought to test the importance of SpoIIE localization at the engulfing membrane in mutants that are miscompartmentalized but where SpoIIIE function is not affected. We resorted to cells lacking SpoIIIM and PbpG which do not affect SpoIIIE stability (20). Previously, we showed that cells lacking *spoIIIM* and *pbpG* exhibit moderate and low miscompartmentalization defects (31% and 6%, respectively) (Fig. 4C) (20). In the Δ*spoIIIM* Δ*pbpG* double mutant miscompartmentalization occurs in over 80% of sporangia (20) (Fig. 4C). Furthermore, combining these mutants with Δ*spoIIQ* results in varying degrees of septal retraction, with the Δ*spoIIIM* Δ*pbpG* Δ*spoIIQ* triple mutant exhibiting septal retraction in >90% of sporangia (20). Thus, we examined miscompartmentalization and septal retraction using the forespore reporter background (P*_spoIIQ_-cfp*) at T3, in Δ*spoIIIM*, Δ*pbpG*, and Δ*spoIIIM* Δ*pbpG* mutants containing SpoIIQ Y28A as the sole source of SpoIIQ (Fig. 4B), and their mutant counterparts that contained Δ*spoIIQ* instead (Fig. 4A).

SpoIIQ Y28A resulted in an increase in miscompartmentalization and septal retraction in Δ*spoIIIM*, Δ*pbpG*, and Δ*spoIIIM* Δ*pbpG* backgrounds relative to the WT, but not to the same extent as Δ*spoIIQ* (Fig. 4C). Quantification showed that in the Δ*spoIIIM* mutant, SpoIIQ Y28A resulted in 21% of sporangia with septal retraction and 36% with miscompartmentalization, while Δ*spoIIQ* resulted in 59.2% of sporangia with septal retraction and 6.9% with miscompartmentalization. In the Δ*pbpG* mutant, SpoIIQ Y28A resulted in 13.9% of sporangia with septal retraction and 12.2% with miscompartmentalization, while Δ*spoIIQ* resulted in 56.2% of sporangia with septal retraction and 14.1% with miscompartmentalization. In the Δ*spoIIIM* Δ*pbpG* double mutant, SpoIIQ Y28A resulted in 65.1% of sporangia with septal retraction and 28.4% with miscompartmentalization, while Δ*spoIIQ* resulted in 96.2% of sporangia with septal retraction and 3.8% with miscompartmentalization.

Since the SpoIIQ Y28A mutant exacerbated the miscompartmentalization and septal retraction defects of Δ*spoIIIM*, Δ*pbpG*, and Δ*spoIIIM* Δ*pbpG*, we would expect to see a decrease in the formation of heat-resistant spores compared to the otherwise WT Δ*spoIIIM*, Δ*pbpG*, and Δ*spoIIIM* Δ*pbpG* mutant counterparts (Fig. 4D). Indeed, SpoIIQ Y28A resulted in a moderate but significant decrease in sporulation efficiency in Δ*spoIIIM* and Δ*pbpG* mutant backgrounds. Sporulation decreased by 29.5% in the Δ*spoIIIM* mutant harboring SpoIIQ Y28A relative to Δ*spoIIIM* (58.4% relative to WT) and by 15.6% in the Δ*pbpG* mutant harboring SpoIIQ Y28A relative to Δ*pbpG* (79.3% relative to WT). In the Δ*spoIIIM* Δ*pbpG* double mutant background, we observed that SpoIIQ Y28A resulted in a slight, but not statistically significant, reduction in sporulation efficiency (2.8% reduction relative to Δ*spoIIIM* Δ*pbpG*). Thus, although SpoIIQ Y28A increases the frequency of septal retraction in the Δ*spoIIIM* Δ*pbpG* mutant background, this does not result in a significant decrease in sporulation. A likely explanation for this observation is that miscompartmentalization is a prerequisite for septal retraction (i.e., only forespores that exhibit miscompartmentalization are subject to retraction) and that some forespores still retain compartmentalization in the Δ*spoIIIM* Δ*pbpG* SpoIIQ Y28A triple mutant—6.5%—similar to the 8.2% heat-resistant spores produced by this mutant (Fig. 4D).

Collectively, these data suggest that the miscompartmentalization and septal retraction observed in the Δ*spoIIQ* mutant are partly due to the mislocalization of SpoIIE. Thus, the localization of SpoIIE to the engulfing membrane by SpoIIQ contributes to septal stabilization at the onset of engulfment.

### Blocking engulfment PG hydrolysis suppresses the miscompartmentalization and septal retraction caused by SpoIIQ Y28A

**Fig 5:**
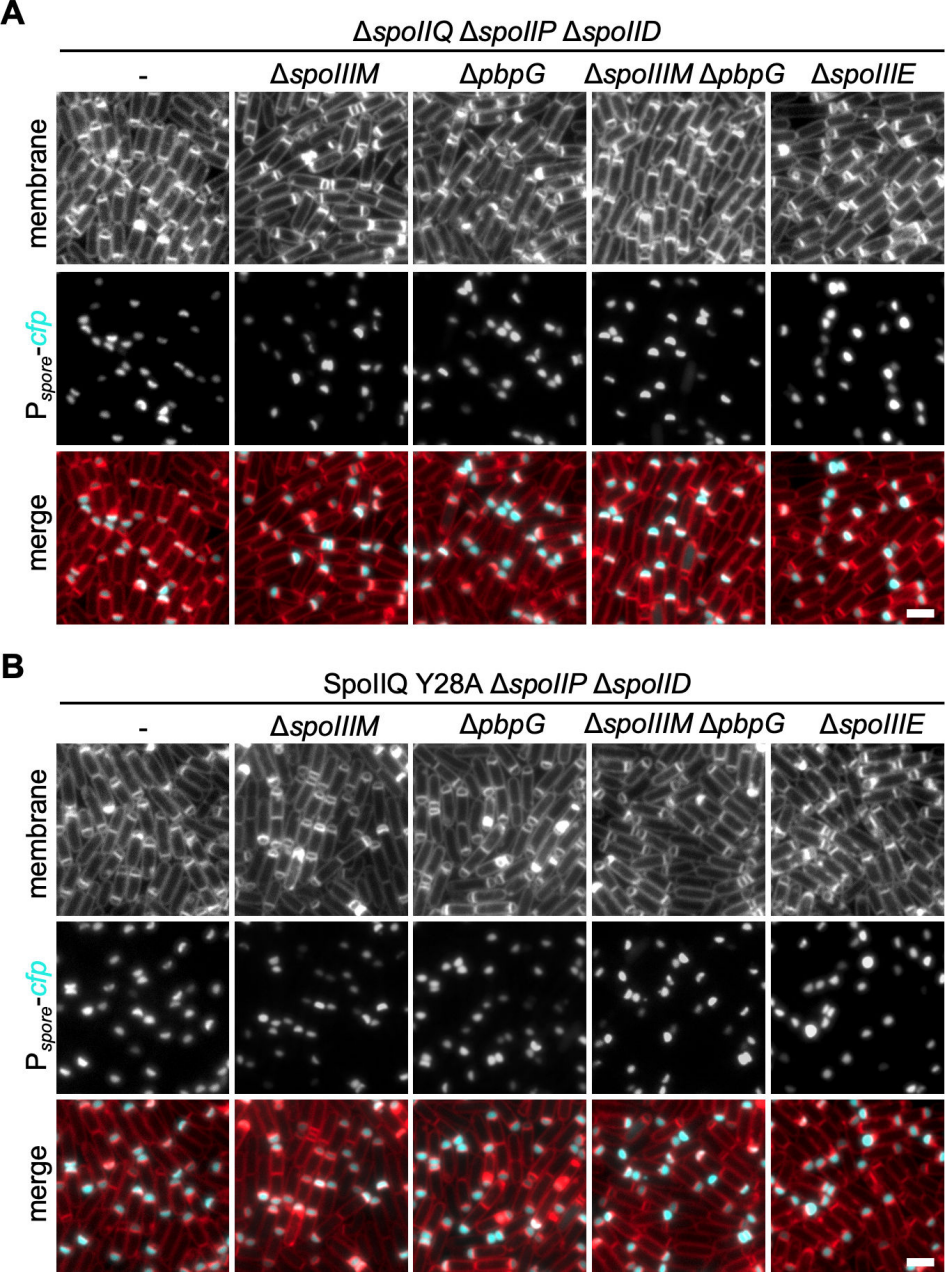
Miscompartmentalization and septal retraction in the SpoIIQ Y28A background are suppressed by blocking septal PG hydrolysis. (**A**) Representative images of septal retraction and miscompartmentalization suppression in various mutants blocked in engulfment in the Δ*spoIIQ* mutant background. Scale bar is 2 µm. (**B**) Representative images of septal retraction and miscompartmentalization suppression in various mutants blocked for engulfment in the SpoIIQ Y28A mutant background. Scale bar is 2 µm.

Septal retraction and miscompartmentalization in the Δ*spoIIQ* background have been shown to occur due to PG hydrolysis by the DMP complex (20). We wanted to know if the septal retraction and miscompartmentalization caused by the SpoIIQ Y28A mutant also depends on PG hydrolysis by this complex. To this end, we introduced deletions of *spoIID* and *spoIIP* into strains where the SpoIIQ Y28A mutant increased septal retraction and miscompartmentalization (as shown in Fig. 2 and 4) and monitored compartmentalization of the forespore-produced CFP reporter at T3 (Fig. 5B). For comparison, and as a matched control, we examined compartmentalization in the Δ*spoIIQ* strains where septal retraction and miscompartmentalization had been shown to occur but is then suppressed with the introduction of *spoIID* and *spoIIP* deletions (20) (Fig. 5A). In the various SpoIIQ Y28A strains lacking Δ*spoIID* Δ*spoIIP*, we observed that the CFP signal was confined to the forespore in virtually all sporangia and was undistinguishable from that observed in the matched strains that contained Δ*spoIIQ* instead. Thus miscompartmentalization and septal retraction in the SpoIIQ Y28A mutant arise due to the activity of the DMP complex. This suggests that relocalization of SpoIIE to the engulfing septal membrane by SpoIIQ functions to counteract the PG hydrolytic activity of the DMP complex.

### The SpoIIIAH-SpoIIQ ratchet plays a minor role in the stabilization of the septum at the onset of engulfment

**Fig 6:**
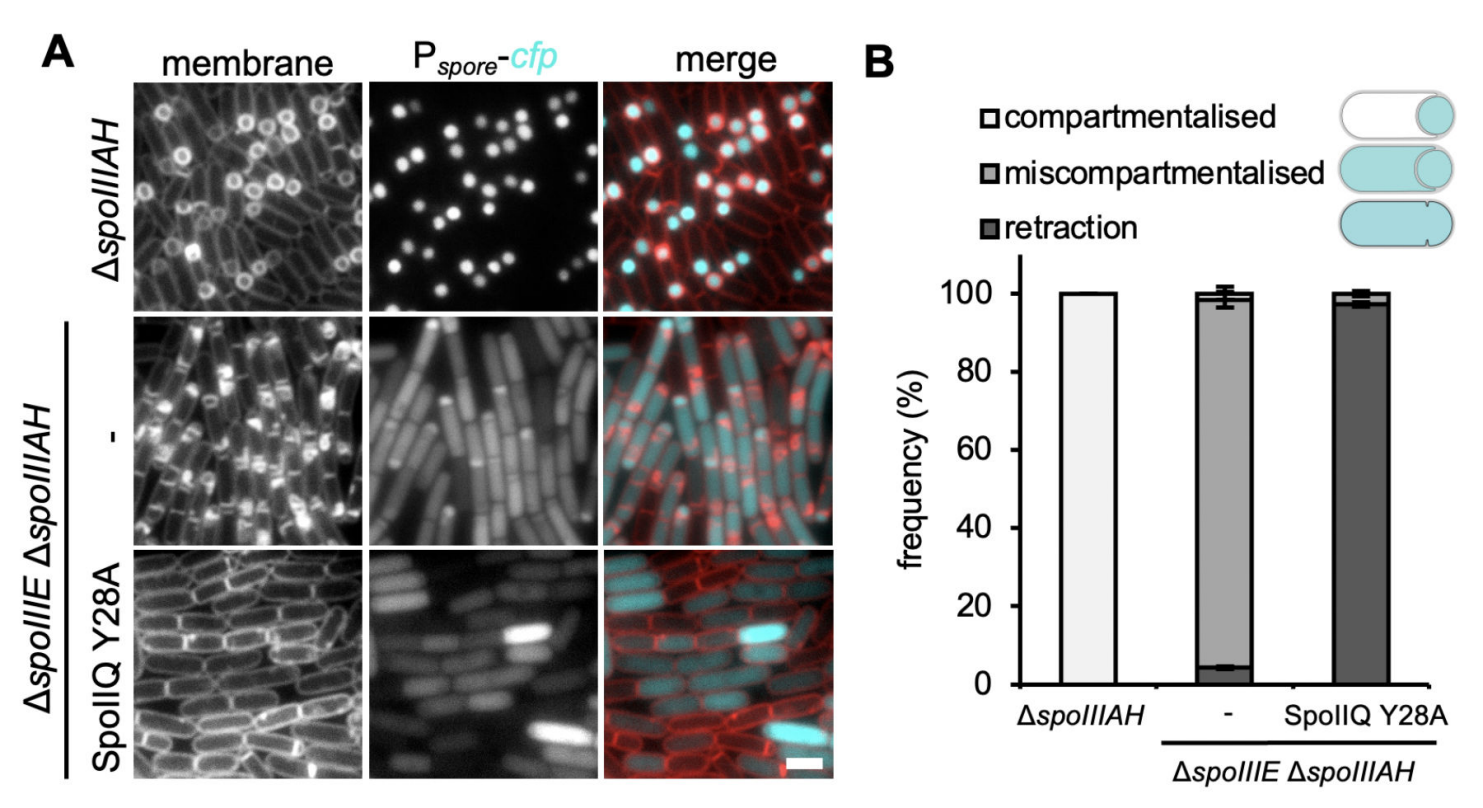
Septal retraction in the absence of SpoIIIAH occurs at a reduced frequency. (**A**) Representative images of the Δ*spoIIIAH* mutant, the Δ*spoIIIE* Δ*spoIIIAH* double mutant, and Δ*spoIIIE* Δ*spoIIIAH* SpoIIQ Y28A triple mutant. Scale bar is 2 µm. (**B**) Histogram showing the average frequency (±SD of three biological replicates, >100 cells per replicate) of compartmentalization, miscompartmentalization, and septal retraction at T3 in the same mutants stated in (**A**).

The initial experiments that defined a role for the SpoIIIAH-SpoIIQ interaction in septal stability were performed in a Δ*spoIIQ* mutant (20), which results in mislocalization of SpoIIIAH and SpoIIE (9, 21, 22). Our results with SpoIIQ Y28A suggest that SpoIIE localization at the engulfing membrane is another pathway in which SpoIIQ contributes to septal stabilization and compartmentalization. Thus, we wanted to know to what degree, if at all, the SpoIIIAH-SpoIIQ intercellular interaction contributes to septal stability and prevention of septal retraction. To this end, we examined septal retraction in a Δ*spoIIIE* Δ*spoIIIAH* double mutant (Fig. 6) and compared it to the Δ*spoIIIE* Δ*spoIIQ* and Δ*spoIIIE* SpoIIQ Y28A double mutants (Fig. 2). Note that in the absence of SpoIIIAH, SpoIIQ remains mostly localized in the engulfing membrane (23), thus allowing us to test the specific role of the SpoIIIAH-SpoIIQ interaction without mislocalizing SpoIIQ. Septal retraction was an infrequent event in the Δ*spoIIIE* Δ*spoIIIAH* double mutant with only 4.3% of sporangia displaying this phenotype (Fig. 6A and B). Interestingly, compared to Δ*spoIIIE*, the Δ*spoIIIE* Δ*spoIIIAH* double mutant displayed a larger proportion of sporangia with severe septal membrane defects and fewer sporangia that had initiated engulfment (Fig. 6A; Fig. S3). Thus while the absence of SpoIIIAH compromises engulfment in the Δ*spoIIIE* mutant, it does not play a major role in maintaining septal membrane stability and preventing septal retraction.

To again demonstrate the importance of SpoIIE localization at the engulfing membrane, we introduced the SpoIIQ Y28A mutation into the Δ*spoIIIE* Δ*spoIIIAH* double mutant background and, as expected, the Δ*spoIIIE* Δ*spoIIIAH* SpoIIQ Y28A triple mutant exhibited septal retraction in virtually all cells (97.1%) (Fig. 6A and B). Finally, blocking DMP complex activity by deleting *spoIID* and *spoIIP* restored compartmentalization in the Δ*spoIIIE* Δ*spoIIIAH* double mutant and Δ*spoIIIE* Δ*spoIIIAH* SpoIIQ Y28A triple mutant (Fig. S4). Collectively, these results indicate that the SpoIIIAH-SpoIIQ ratchet plays a minor role in septal stabilization at the onset of engulfment.

## DISCUSSION

Although the interaction between SpoIIQ and SpoIIE has been known for over 15 years (9), its exact significance has remained mysterious. The discovery of a SpoIIQ mutant, SpoIIQ Y28A, that results in SpoIIE mislocalization in the forespore (10), opened the doors for further dissection of the significance of this interaction. Building on a new role we recently discovered for SpoIIQ in septal stabilization and compartmentalization at the onset of engulfment, we utilized the SpoIIQ Y28A mutant to probe a role for SpoIIE in these processes. We show, using various mutants with defects in septal stabilization and compartmentalization, that SpoIIQ-mediated localization of SpoIIE to the engulfing membrane plays an important role in ensuring septal stability and compartmentalization at the onset of engulfment. Thus, we have identified an additional role for the essential and highly conserved SpoIIE protein in the engulfment stage of spore development.

The increased septal retraction and miscompartmentalization frequency in the SpoIIQ Y28A mutant, when other septal stability factors are missing (such as Δ*spoIIIE*, Δ*spoIIIM*, and Δ*pbpG*), suggests that SpoIIQ-dependent SpoIIE localization contributes to septal stability at the onset of engulfment. Miscompartmentalization and septal retraction are thought to occur when PG hydrolysis by the DMP complex at the septum is not balanced with PG synthesis and when stabilization of the septum is reduced due to diminished interactions between septal proteins and septal PG (20). Interestingly, bacterial two-hybrid and *in vivo* co-immunoprecipitation assays suggest that SpoIIE interacts with proteins involved in PG synthesis and this includes RodZ, DivIVA, EzrA, GpsB, Pbp1a (PonA), Pbp2a (PbpA), Pbp2b (PbpB), and Pbp4b (PbpI) (24–26). Furthermore, it appears that GpsB co-localizes with SpoIIE in the engulfing membrane (24). Although it remains unclear if the interactions between SpoIIE and all the aforementioned proteins occur in the engulfing membrane, we hypothesize that SpoIIE contributes to maintaining the balance between PG synthesis and PG hydrolysis early in this process. SpoIIE localization at the engulfing membrane likely facilitates the retention of the PG synthetic machinery there to ensure septal stabilization upon PG hydrolysis by the DMP complex. Consistent with the idea that SpoIIE reinforces septal stability at the onset of engulfment by contributing to PG synthesis, we observed that blocking PG hydrolysis by the DMP complex suppressed the septal stability and miscompartmentalization defects observed in the SpoIIQ Y28A mutant (Fig. 5).

Interestingly, while SpoIIQ and SpoIIQ Y28 are required for enrichment of SpoIIE in the engulfing membrane (Fig. 1), our analysis suggests that in the SpoIIQ Y28A mutant, and to a lesser degree in the Δ*spoIIQ* mutant, SpoIIE has the propensity to localize ahead of the engulfing membrane (Fig. S1). The significance of this observation remains unclear. However, it has been shown that some proteins involved in PG synthesis, namely PbpB, PbpC, PonA, and MreB also localize ahead of the engulfing membrane when expressed from a σF-dependent promoter (12). The localization of these proteins at that position has led to a model where PG synthesis ahead of the engulfing membrane contributes to efficient engulfment by providing a substrate for PG hydrolysis by the DMP complex (12). Thus, by interacting simultaneously with SpoIIQ, and proteins of the PG synthetic machinery, SpoIIE may function to concentrate-specific PG synthases to the septal membrane at the onset of engulfment. Identifying which component or components of the PG synthetic machinery contribute to septal stabilization and how SpoIIE interacts with these components, could reveal the finer mechanistic details of the role of SpoIIE in governing PG synthesis at the early stages of engulfment.

Our results with SpoIIQ Y28A force us to return to the proposed role of the SpoIIIAH-SpoIIQ ratchet in maintaining septal stability and preventing septal retraction (20). The initial experiments that defined a role for the SpoIIIAH-SpoIIQ ratchet in septal stability were performed in a Δ*spoIIQ* mutant (20). These experiments left open the possibility that either of SpoIIQ’s known interactions contributes to septal stability. Our data suggest that the SpoIIQ-SpoIIE interaction plays a more important role in septal stability and prevention of septal retraction than the SpoIIIAH-SpoIIQ interaction (Fig. 2 and 6). Indeed, septal retraction was higher in the Δ*spoIIIE* SpoIIQ Y28A double mutant than in the Δ*spoIIIE* Δ*spoIIIAH* double mutant (Fig. 2 and 6). Consequently, it can be inferred that the SpoIIIAH-SpoIIQ interaction, while important in maintaining engulfment efficiency through the proposed biophysical ratchet (13), plays little role in septal stabilization at the onset of engulfment. Interestingly, our data suggest that SpoIIQ itself, or its unknown interaction(s) partners, also play a role in septal stability. This is illustrated in the comparison of septal retraction frequency in the different double mutants: Δ*spoIIIE* Δ*spoIIIAH* (4.3%), Δ*spoIIIE* SpoIIQ Y28A (62.4%), and Δ*spoIIIE* Δ*spoIIQ* (97.7%) with the latter exhibiting the highest frequency. We also tested the role of GerM, a mother cell protein that requires SpoIIQ for its localization and partly influences SpoIIQ localization (23). However, the Δ*spoIIIE* Δ*gerM* double mutant was comparable to Δ*spoIIIE* in terms of septal retraction (Fig. S5). Identifying the full set of SpoIIQ interacting proteins that contribute to septal stability remains a challenge for the future.

How SpoIIQ interacts with SpoIIE remains an outstanding question. Since amino acid tyrosine 28 (Y28) in SpoIIQ’s transmembrane helix is critical for the localization of SpoIIE to the engulfing membrane (10), we hypothesize that there are likely residues in one of SpoIIE’s 10 transmembrane helices that establish an interaction with SpoIIQ Y28, or with other residues located in the SpoIIQ transmembrane helix. Identifying the molecular basis of the SpoIIQ-SpoIIE interaction could reveal additional insights into how these proteins contribute to septal stability at the onset of engulfment.

**Fig 7:**
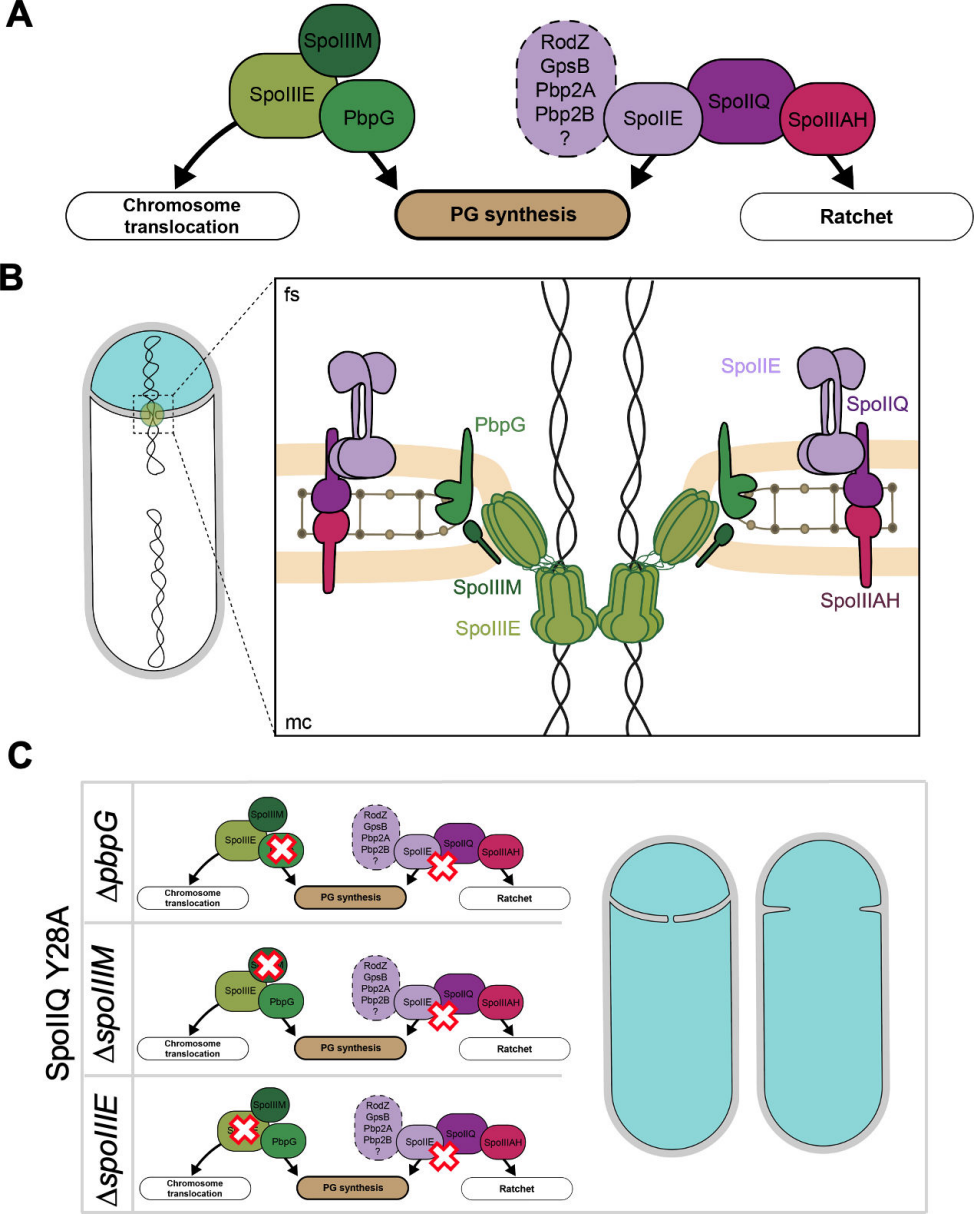
Septal stabilization at the onset of engulfment occurs through two pathways connected to peptidoglycan synthesis, the SpoIIIE and SpoIIQ pathway. (**A**) Diagram illustrating the two pathways that contribute to peptidoglycan synthesis at the onset of engulfment, with the SpoIIIE pathway in green tones and the SpoIIQ pathway in purple tones. The SpoIIIE pathway involves PbpG and SpoIIIM. The SpoIIQ pathway involves SpoIIE and its interactions with proteins involved in peptidoglycan synthesis that likely contribute to septal stabilization. (**B**) Schematic illustrating the septal pore where these two pathways contribute to septal stabilization and compartmentalization (fs, forespore; mc, mother cell). (**C**) Diagram summarizing the major genetic backgrounds that support a role for SpoIIE in compartmentalization and septal stabilization at the onset of engulfment. The red delineated crosses illustrate how mutating one protein of the SpoIIIE pathway and another protein in the SpoIIQ pathway (SpoIIQ Y28A) results in increased miscompartmentalization and septal retraction.

Based on the above and what is already known about the maintenance of septal stability and compartmentalization during the early stages of engulfment (20), we propose that there are two major pathways connected to PG synthesis that contribute to these processes, the SpoIIQ and SpoIIIE pathway (Fig. 7). The SpoIIQ pathway involves SpoIIE and the likely interactions it establishes with the PG synthetic machinery (24, 25), and the SpoIIIE pathway that involves SpoIIIM and PbpG (20). Together, these pathways ensure septal stability and compartmentalization at a critical time in development where there is simultaneous remodeling of the septum and chromosome translocation into the forespore through a pore in this septum. Finally, this work highlights the complexity of the protein networks during sporulation and how highly conserved proteins, like SpoIIE, SpoIIQ, and SpoIIIE orchestrate various morphogenetic processes during spore development.

## MATERIALS AND METHODS

### General methods

All *Bacillus subtilis* strains originated from the auxotrophic strain 168 (Table S1). Sporulation induction was achieved through resuspension at 37°C following the Sterlini-Mandelstam method (27) or by nutrient depletion in a supplemented Difco sporulation medium (DSM) (28) consisting of 8 g/L Bacto nutrient broth (Difco), 0.1% (wt/vol) KCl, 1 mM MgSO_4_, 0.5 mM NaOH, 1 mM Ca(NO_3_)_2_, 0.01 mM MnCl_2_, and 0.001 mM FeSO_4_. Sporulation efficiency was determined through the heat-kill assay conducted on cultures grown for 30 hours at 37°C in the DSM medium. The total number of heat-resistant (80°C for 20 minutes) CFUs (colony-forming units) is compared with wild-type heat-resistant CFUs.

### Fluorescence microscopy

Live-cell fluorescence imaging was conducted by placing cells on a 2% (wt/vol) agarose pad prepared using resuspension medium and set with a Gene Frame (Bio-Rad). For sporulating cell cultures prepared using the resuspension method, when a predetermined time point was reached, 250 µL of culture was pelleted via centrifugation and then resuspended in 10 µL of resuspension medium containing 0.05 mM TMA-DPH [1-(4-trimethylammoniumphenyl)−6-phenyl-1,3,5-hexatriene p-toluenesulfonate]. Subsequently, 2 µL of cell suspension was spread onto an agarose pad, and a coverslip was placed on top of the Gene Frame. Cells were imaged using a Zeiss Axio Observer 7 microscope equipped with a Plan-Apochromat 100×/1.4 Oil Ph3 objective and a Colibri 7 Type R[G/Y]CBV-UV fluorescent light source. Images were captured with an Axiocam 712 mono camera. The TMA-DPH membrane dye was excited with a Zeiss Axio 92HE filter, using an exposure time of 100 ms. CFP was excited with a Zeiss Axio 108HE filter with an exposure time of 150 ms. YFP was excited with a Zeiss Axio 108HE filter with an exposure time of 300 ms.

SIM was conducted on the Zeiss Elyra 7, a wide-field-based high-resolution system, equipped with a PCO edge camera (pco.edge 4.2 sCMOS camera). Coherent lasers emitting at 488 nm (100 mW) and 561 nm (100 mW) were used to excite SpoIIE-YFP and the FM4-64 membrane dye, respectively. The exposure time was 80 ms.

### Image analysis and statistics

Post-processing of images was conducted by adjusting brightness, and contrast with the Fiji software (version 2.14.0/1.54f) (29). Quantitative analyses were conducted by applying the manual counting tool within the Fiji software, and then raw data were exported to Excel (Microsoft, version 16.83) for data compilation and graph generation. For quantification of the SpoIIE-YFP localization phenotypes (Fig. 1C), “enriched” SpoIIE localization was defined as YFP signal that was localized in the engulfing membrane (curving or curved septal membrane), and “non-enriched” was defined as YFP signal that was present but not localized in the engulfing membrane (curving or curved septal membrane). Enriched and non-enriched frequencies were calculated relative to the total number of cells displaying YFP signal and a curving or curved septal membrane.

For quantification of the miscompartmentalization defect, any cell exhibiting a CFP signal originating from the forespore in the mother cell that had a visible spore compartment (including deformed spores) was considered miscompartmentalized. For quantification of septal retraction, cells that displayed a CFP signal originating from the forespore in the mother cell, accompanied by a non-continuous polar septum or no signs of a polar septum, were considered to have a retracted septum. In both cases, miscompartmentalization and septal retraction frequency were calculated relative to the total number of cells displaying CFP signal (i.e. cells that had activated σF).

For the quantification of SpoIIE-YFP signal in the engulfing membrane (Fig. S1B and C), background subtracted images were used. Next, the Fiji “Straight Line” tool at a width thickness of 4 was used to draw a line across the forespore membranes, starting in the mother cell cytoplasm and ending outside the forespore. Next, the “Plot Profile” tool was used to populate both the TMA-DPH membrane dye and SpoIIE-YFP signal intensity value across the line. The line length used was the same for all forespores examined and resulted in the collection of 39 signal intensity values per line. Next, the data were exported to Excel (Microsoft, version 16.83), and the data were normalized using the “standardized function”. The normalized data for the TMA-DPH membrane dye signal intensity and SpoIIE-YFP signal intensity was then populated to generate the line plots shown in Fig. S1B.

The Student’s *t*-test and one-way analysis of variance were used to compare the means (three biological replicates) of two groups stated in the figure legends.

## SUPPLEMENTAL MATERIAL

Supplemental figures jb.00220-24-s0001.pdf Figures S1 to S5.

Table S1 jb.00220-24-s0002.pdf Strain list and plasmid construction.

ASM does not own the copyrights to Supplemental Material that may be linked to, or accessed through, an article. The authors have granted ASM a non-exclusive, world-wide license to publish the Supplemental Material files. Please contact the corresponding author directly for reuse.

## References

[1] Molle V, Fujita M, Jensen ST, Eichenberger P, González-Pastor JE, Liu JS, Losick R. 2003. The Spo0A regulon of Bacillus subtilis. Mol Microbiol 50:1683–1701. doi:10.1046/j.1365-2958.2003.03818.x 14651647

[2] Fujita M, Losick R. 2005. Evidence that entry into sporulation in Bacillus subtilis is governed by a gradual increase in the level and activity of the master regulator Spo0A. Genes Dev 19:2236–2244. doi:10.1101/gad.1335705 16166384 PMC1221893

[3] Piggot PJ, Hilbert DW. 2004. Sporulation of Bacillus subtilis. Curr Opin Microbiol 7:579–586. doi:10.1016/j.mib.2004.10.001 15556029

[4] Dworkin J, Losick R. 2005. Developmental commitment in a bacterium. Cell 121:401–409. doi:10.1016/j.cell.2005.02.032 15882622

[5] Carniol K, Ben-Yehuda S, King N, Losick R. 2005. Genetic dissection of the sporulation protein SpoIIE and its role in asymmetric division in Bacillus subtilis. J Bacteriol 187:3511–3520. doi:10.1128/JB.187.10.3511-3520.2005 15866939 PMC1112011

[6] Barák I, Behari J, Olmedo G, Guzmán P, Brown DP, Castro E, Walker D, Westpheling J, Youngman P. 1996. Structure and function of the Bacillus SpoIIE protein and its localization to sites of sporulation septum assembly. Mol Microbiol 19:1047–1060. doi:10.1046/j.1365-2958.1996.433963.x 8830262

[7] Bradshaw N, Losick R. 2015. Asymmetric division triggers cell-specific gene expression through coupled capture and stabilization of a phosphatase. Elife 4:e08145. doi:10.7554/eLife.08145 26465112 PMC4714977

[8] Khanna K, Lopez-Garrido J, Sugie J, Pogliano K, Villa E. 2021. Asymmetric localization of the cell division machinery during Bacillus subtilis sporulation. Elife 10:e62204. doi:10.7554/eLife.62204 34018921 PMC8192124

[9] Campo N, Marquis KA, Rudner DZ. 2008. SpoIIQ anchors membrane proteins on both sides of the sporulation septum in Bacillus subtilis. J Biol Chem 283:4975–4982. doi:10.1074/jbc.M708024200 18077456

[10] Flanagan KA, Comber JD, Mearls E, Fenton C, Wang Erickson AF, Camp AH. 2016. A membrane-embedded amino acid couples the SpoIIQ channel protein to anti-sigma factor transcriptional repression during Bacillus subtilis sporulation. J Bacteriol 198:1451–1463. doi:10.1128/JB.00958-15 26929302 PMC4836239

[11] Khanna K, Lopez-Garrido J, Zhao Z, Watanabe R, Yuan Y, Sugie J, Pogliano K, Villa E. 2019. The molecular architecture of engulfment during Bacillus subtilis sporulation. Elife 8:e45257. doi:10.7554/eLife.45257 31282858 PMC6684271

[12] Ojkic N, López-Garrido J, Pogliano K, Endres RG. 2016. Cell-wall remodeling drives engulfment during Bacillus subtilis sporulation. Elife 5:e18657. doi:10.7554/eLife.18657 27852437 PMC5158138

[13] Ojkic N, López-Garrido J, Pogliano K, Endres RG. 2014. Bistable forespore engulfment in Bacillus subtilis by a zipper mechanism in absence of the cell wall. PLoS Comput Biol 10:e1003912. doi:10.1371/journal.pcbi.1003912 25356555 PMC4214620

[14] Riley EP, Schwarz C, Derman AI, Lopez-Garrido J. 2020. Milestones in Bacillus subtilis sporulation research. Microb Cell 8:1–16. doi:10.15698/mic2021.01.739 33490228 PMC7780723

[15] Morlot C, Uehara T, Marquis KA, Bernhardt TG, Rudner DZ. 2010. A highly coordinated cell wall degradation machine governs spore morphogenesis in Bacillus subtilis. Genes Dev 24:411–422. doi:10.1101/gad.1878110 20159959 PMC2816739

[16] Chastanet A, Losick R. 2007. Engulfment during sporulation in Bacillus subtilis is governed by a multi‐protein complex containing tandemly acting autolysins. Mol Microbiol 64:139–152. doi:10.1111/j.1365-2958.2007.05652.x 17376078

[17] Chan H, Taib N, Gilmore MC, Mohamed AMT, Hanna K, Luhur J, Nguyen H, Hafiz E, Cava F, Gribaldo S, Rudner D, Rodrigues CDA. 2022. Genetic screens identify additional genes implicated in envelope remodeling during the engulfment stage of Bacillus subtilis sporulation. mBio 13:e0173222. doi:10.1128/mbio.01732-22 36066101 PMC9600426

[18] Broder DH, Pogliano K. 2006. Forespore engulfment mediated by a ratchet-like mechanism. Cell 126:917–928. doi:10.1016/j.cell.2006.06.053 16959571 PMC3266857

[19] Chan H, Mohamed AMT, Grainge I, Rodrigues CDA. 2022. FtsK and SpoIIIE, coordinators of chromosome segregation and envelope remodeling in bacteria. Trends Microbiol 30:480–494. doi:10.1016/j.tim.2021.10.002 34728126

[20] Mohamed AMT, Chan H, Luhur J, Bauda E, Gallet B, Morlot C, Cole L, Awad M, Crawford S, Lyras D, Rudner DZ, Rodrigues CDA. 2021. Chromosome segregation and peptidoglycan remodeling are coordinated at a highly stabilized septal pore to maintain bacterial spore development. Dev Cell 56:36–51. doi:10.1016/j.devcel.2020.12.006 33383000 PMC8048138

[21] Blaylock B, Jiang X, Rubio A, Moran CP, Pogliano K. 2004. Zipper-like interaction between proteins in adjacent daughter cells mediates protein localization. Genes Dev 18:2916–2928. doi:10.1101/gad.1252704 15574594 PMC534652

[22] Doan T, Marquis KA, Rudner DZ. 2005. Subcellular localization of a sporulation membrane protein is achieved through a network of interactions along and across the septum. Mol Microbiol 55:1767–1781. doi:10.1111/j.1365-2958.2005.04501.x 15752199

[23] Rodrigues CDA, Ramírez-Guadiana FH, Meeske AJ, Wang X, Rudner DZ. 2016. GerM is required to assemble the basal platform of the SpoIIIA–SpoIIQ transenvelope complex during sporulation in Bacillus subtilis. Mol Microbiol 102:260–273. doi:10.1111/mmi.13457 27381174 PMC5055438

[24] Muchová K, Chromiková Z, Barák I. 2020. Linking the peptidoglycan synthesis protein complex with asymmetric cell division during Bacillus subtilis sporulation. Int J Mol Sci 21:12. doi:10.3390/ijms21124513 PMC7349982 32630428

[25] Muchová K, Chromiková Z, Bradshaw N, Wilkinson AJ, Barák I. 2016. Morphogenic protein RodZ interacts with sporulation specific SpoIIE in Bacillus subtilis. PLoS One 11:e0159076. doi:10.1371/journal.pone.0159076 27415800 PMC4945075

[26] Eswaramoorthy P, Winter PW, Wawrzusin P, York AG, Shroff H, Ramamurthi KS. 2014. Asymmetric division and differential gene expression during a bacterial developmental program requires DivIVA. PLoS Genet 10:e1004526. doi:10.1371/journal.pgen.1004526 25101664 PMC4125091

[27] Sterlini JM, Mandelstam J. 1969. Commitment to sporulation in Bacillus subtilis and its relationship to development of actinomycin resistance. Biochem J 113:29–37. doi:10.1042/bj1130029 4185146 PMC1184601

[28] Schaeffer P, Millet J, Aubert J-P. 1965. Catabolic repression of bacterial sporulation. Proc Natl Acad Sci U S A 54:704–711. doi:10.1073/pnas.54.3.704 4956288 PMC219731

[29] Schindelin J, Arganda-Carreras I, Frise E, Kaynig V, Longair M, Pietzsch T, Preibisch S, Rueden C, Saalfeld S, Schmid B, Tinevez J-Y, White DJ, Hartenstein V, Eliceiri K, Tomancak P, Cardona A. 2012. Fiji: an open-source platform for biological-image analysis. Nat Methods 9:676–682. doi:10.1038/nmeth.2019 22743772 PMC3855844

