# Supplementary material for: SpoIIQ-dependent localization of SpoIIE contributes to septal stability and compartmentalization during the engulfment stage of Bacillus subtilis sporulation
Source: J Bacteriol. 2024 Jun 21;206(7):e00220-24. doi: 10.1128/jb.00220-24 (PMC11270862; doi:10.1128/jb.00220-24)
Supplement: Supplemental figures — Figures S1 to S5. [file jb.00220-24-s0001.pdf]

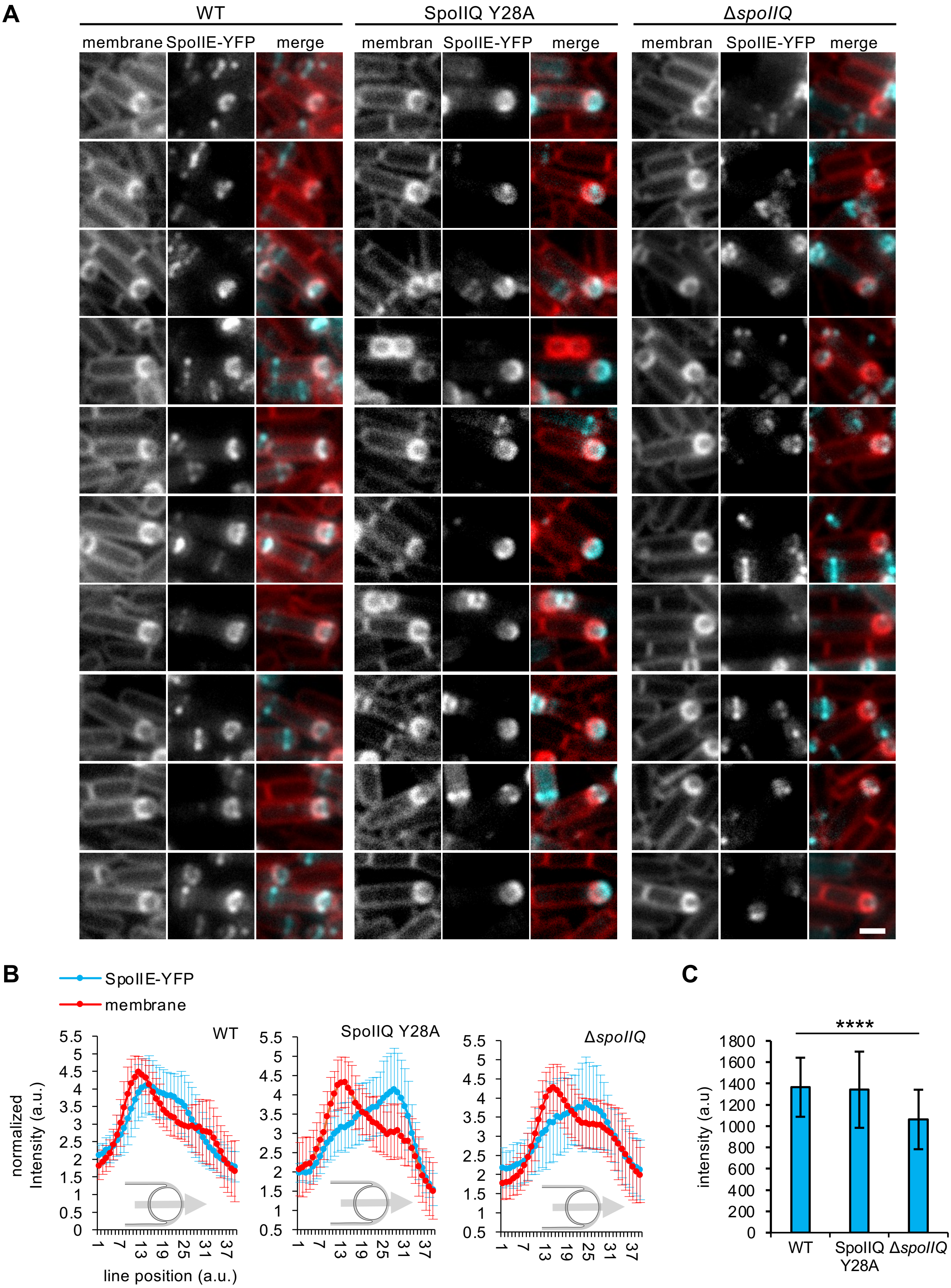

Figure S1. Representative images and quantification of SpoIIE-YFP signal intensity across the engulfing membrane. (A) Representative zoomed-in examples of SpoIIE-YFP localisation in WT, the Δ*spoIIQ* mutant and the SpoIIQ Y28A mutant. SpoIIE-YFP is pseudocoloured in cyan. Scale bar is 1 µm. **(B)** Normalised intensity (mean ± SD, n=10) of the TMA-DPH membrane dye and SpoIIE-YFP signal across the forespore membranes, in WT, the Δ*spoIIQ* mutant and SpoIIQ Y28A mutant. **(C)** Quantification of SpoIIE-YFP intensity (mean ± SD, n=10) in WT, the SpoIIQ Y28A mutant and Δ*spoIIQ* mutant. ****, $p < 0.0001$, by Student's t-test and one-way ANOVA.

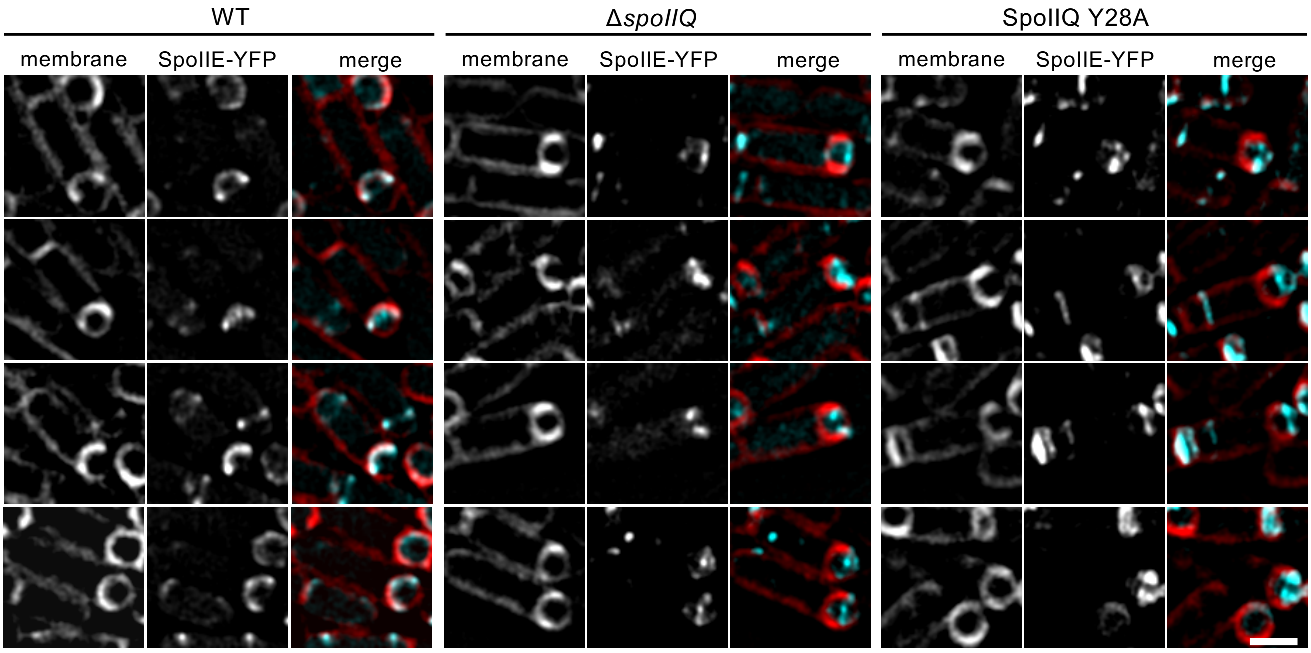

Figure S2. SpoIIE-YFP localisation visualised using SIM. Representative zoomed-in examples of SpoIIE-YFP localisation in WT, the Δ*spoIIQ* mutant and the SpoIIQ Y28A mutant. In WT cells, SpoIIE-YFP tracks the engulfing membrane. In the Δ*spoIIQ* and in the SpoIIQ Y28A mutants, SpoIIE-YFP is not enriched in the engulfing membrane and appears to localize ahead of the engulfing membrane. SpoIIE-YFP is pseudocoloured in cyan. Scale bar is 1 μm.

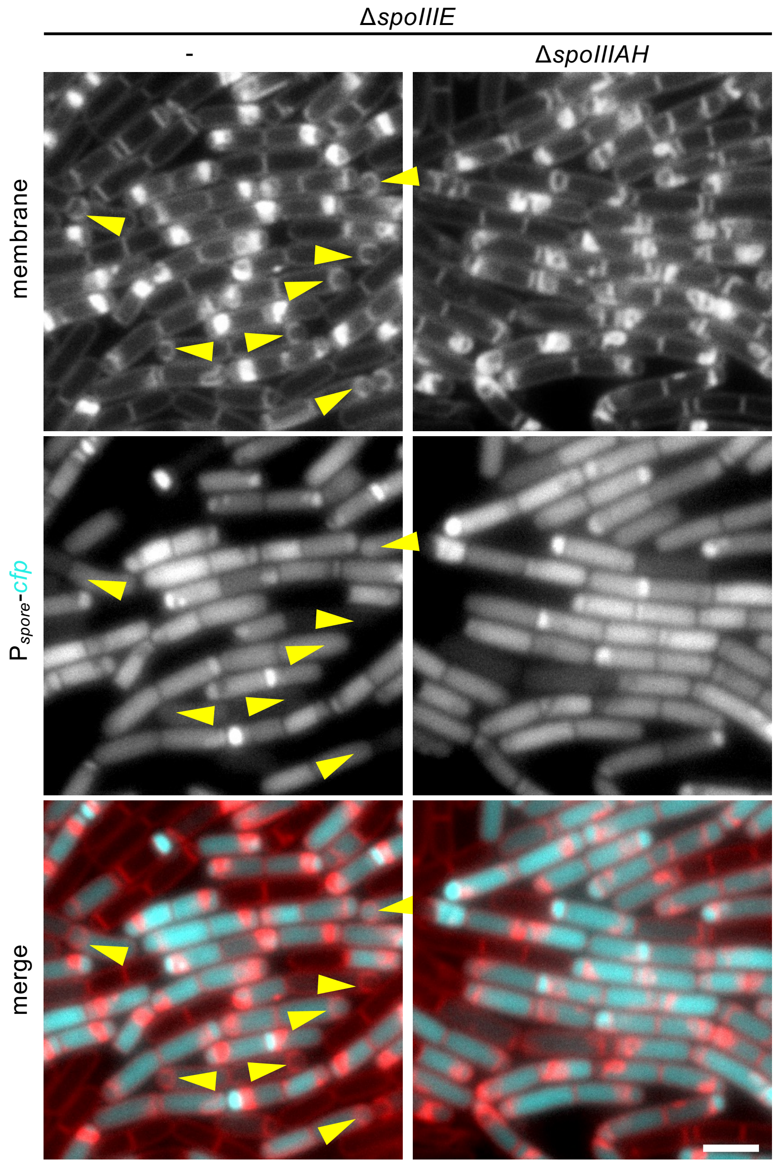

Figure S3: Representative images of the morphological defects in the Δ*spoIIIE* mutant and Δ*spoIIIE* Δ*spoIIIH* double mutant. While both mutants exhibit membrane blebs and deformities in the septal membrane, the Δ*spoIIIE* mutant has a higher proportion of cells that initiate engulfment (yellow arrowheads). Images were collected at T3. Scale bar is 2 µm.

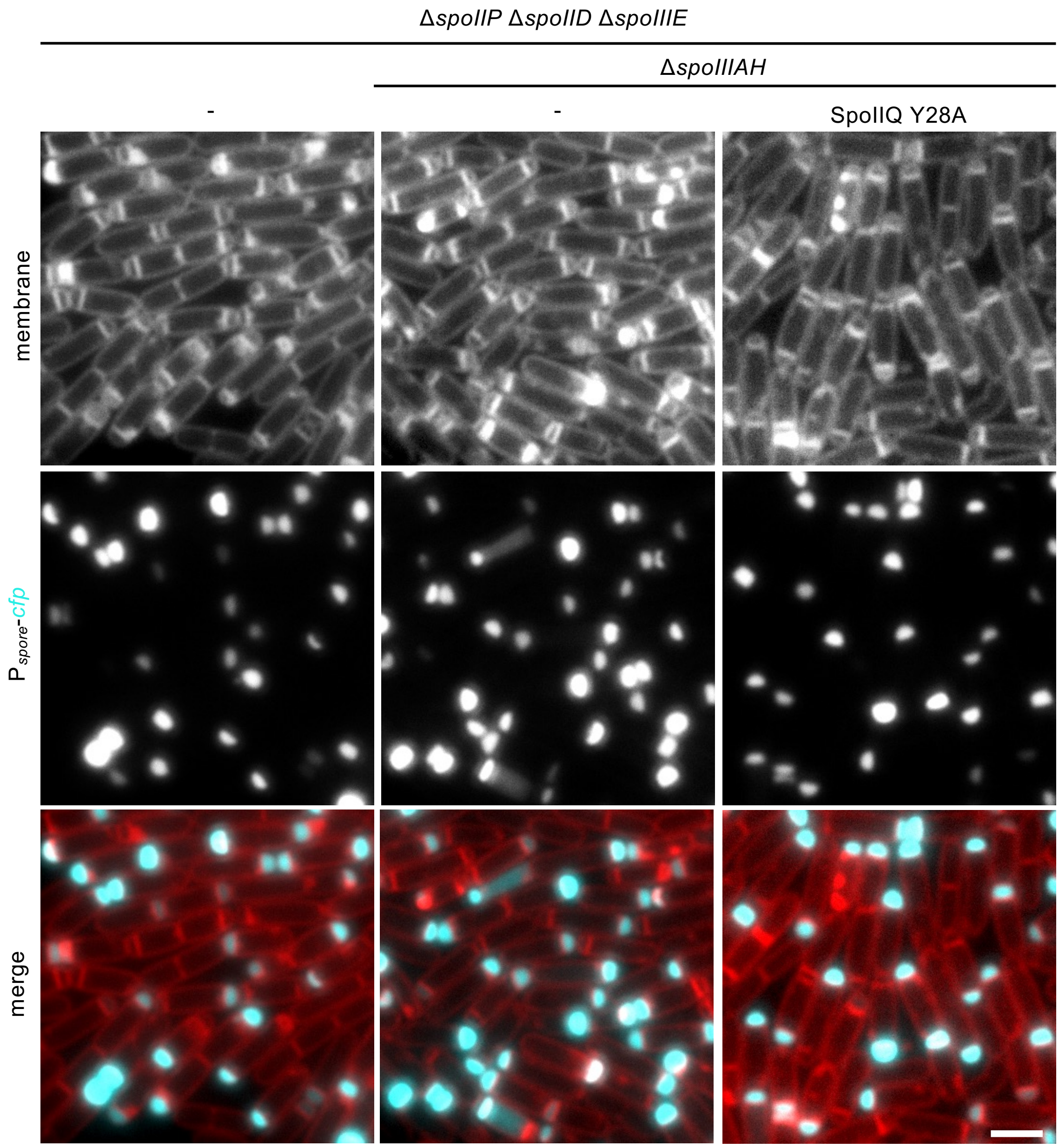

Figure S4. Miscompartmentalisation and septal retraction in the Δ*spoIIIAH* SpoIIQ Y28A double mutant background is suppressed by blocking septal PG hydrolysis. Representative images of septal retraction and miscompartmentalisation suppression in the Δ*spoIIP* Δ*spoIID* Δ*spoIIIE* mutants. Suppression of retraction and msiscompartmentalization also occurs in the Δ*spoIIP* Δ*spoIID* Δ*spoIIIE* Δ*spoIIIAH* quadruple mutant and Δ*spoIIP* Δ*spoIID* Δ*spoIIIE* Δ*spoIIIAH* SpoIIQ Y28A quintuple mutant. Scale bar is 2 µm.

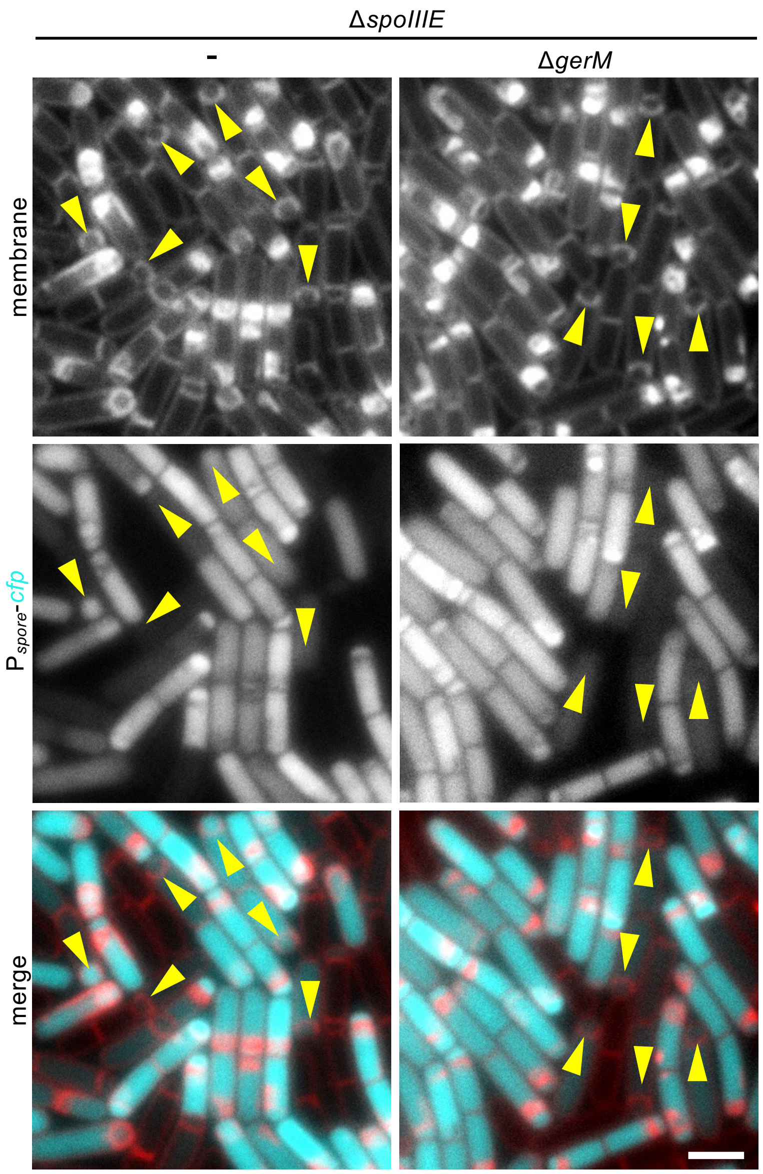

Figure S5: Representative images of the morphological defects of the Δ*spoIIIE* mutant and Δ*spoIIIE* Δ*gerM* double mutant. Images were collected at T3. Like the Δ*spoIIIE* mutant, engulfment is observed in some Δ*spoIIIE* Δ*gerM* mutant cells (yellow arrowheads). Scale bar is 2 μm.
