# Supplementary material for: SpoIIQ-dependent localization of SpoIIE contributes to septal stability and compartmentalization during the engulfment stage of Bacillus subtilis sporulation
Source: J Bacteriol. 2024 Jun 21;206(7):e00220-24. doi: 10.1128/jb.00220-24 (PMC11270862; doi:10.1128/jb.00220-24)
Supplement: Table S1 — Strain list and plasmid construction. [file jb.00220-24-s0002.pdf]

SUPPLEMENTARY MATERIAL

Table S1: Strains and plasmids used in this study.

Construct	Genotype/description	Source
B. subtilis strains		
bAT87	*amyE::PspoIIQ-cfp(Bs) (cat)*	*(Mohamed et al. 2022)*
bAT88	*amyE::PspoIIQ-cfp(Bs) (cat), spoIIIM::erm*	*(Mohamed et al. 2022)*
bAT89	*amyE::PspoIIQ-cfp(Bs) (cat), pbpG::kan*	*(Mohamed et al. 2022)*
bAT91	*amyE::PspoIIQ-cfp(Bs) (cat), pbpG::kan, spoIIIM::erm*	*(Mohamed et al. 2022)*
bAT213	*amyE::PspoIIQ-cfp(Bs) (cat), spoIIIAH::spec*	*(Mohamed et al. 2022)*
bAT457	*amyE::PspoIIQ-cfp(Bs) (cat), spoIIIE::neo*	*(Mohamed et al. 2022)*
bAT478	*amyE::PspoIIQ-cfp(Bs) (cat), spoIIQ::erm*	*(Mohamed et al. 2022)*
bAT479	*amyE::PspoIIQ-cfp(Bs) (cat), spoIIQ::erm, spoIIIM::lox72*	*(Mohamed et al. 2022)*
bAT480	*amyE::PspoIIQ-cfp(Bs) (cat), pbpG::lox72, spoIIQ::erm*	*(Mohamed et al. 2022)*
bAT481	*amyE::PspoIIQ-cfp(Bs) (cat), pbpG::lox72, spoIIQ::erm, spoIIIM::lox72*	*(Mohamed et al. 2022)*
bAT491	*amyE::PspoIIQ-cfp(Bs) (cat), spoIID::spec, spoIIP::tet spoIIQ::erm, spoIIIM::lox72*	*(Mohamed et al. 2022)*
bAT492	*amyE::PspoIIQ-cfp(Bs) (cat), pbpG::lox72, spoIIP::tet, spoIID::spec, spoIIQ::erm*	*(Mohamed et al. 2022)*
bAT493	*amyE::PspoIIQ-cfp(Bs) (cat), pbpG::lox72, spoIID::spec, spoIIP::tet spoIIQ::erm, spoIIIM::lox72*	*(Mohamed et al. 2022)*
bAT705	*amyE::PspoIIQ-cfp(Bs) (cat), spoIIIE::kan*	*(Mohamed et al. 2022)*
bBD017	*amyE::PspoIIQ-cfp(cat), spoIIIE:: lox72, ycgO::PspoIIIE-spoIIIE*D584A (phleo)*	This study
bBD046	*amyE::PspoIIQ-cfp(Bs) (cat), spoIIIE::lox72, spoIIQ::erm*	This study
bBD085	*amyE::PspoIIQ-cfp(Bs) (cat), spoIIEΩpKM282a(spoIIE-yfp(spec))*	This study
bBD086	*amyE::PspoIIQ-cfp(Bs) (cat), spoIIEΩpKM282a(spoIIE-yfp(spec)), spoIIQ::erm*	This study
bBD105	*amyE::PspoIIQ-cfp(Bs) (cat), spoIIQ::erm, ycgO::PspoIIQ-spoIIQ Y28A (kan), spoIIIM:: lox72*	This study
bBD106	*amyE::PspoIIQ-cfp(Bs) (cat), pbpG:: lox72, spoIIQ::erm, ycgO::PspoIIQ-spoIIQ Y28A (kan)*	This study
bBD108	*amyE::PspoIIQ-cfp(Bs) (cat), pbpG::lox72, spoIIQ::erm, ycgO::PspoIIQ-spoIIQ Y28A (kan), spoIIIM:: lox72.*	This study
bBD110	*amyE::PspoIIQ-cfp(Bs) (cat), spoIID::spec, spoIIP::tet spoIIQ::erm, ycgO::PspoIIQ-spoIIQ Y28A (kan), spoIIIM:: lox72*	This study
bBD111	*amyE::PspoIIQ-cfp(Bs) (cat), pbpG:: lox72, spoIID::spec, spoIIP::tet, spoIIQ::erm, ycgO::PspoIIQ-spoIIQ Y28A (kan)*	This study
bBD112	*amyE::PspoIIQ-cfp(Bs) (cat), pbpG:: lox72, spoIID::spec, spoIIP::tet spoIIQ::erm, ycgO::PspoIIQ-spoIIQ Y28A (kan), spoIIIM:: lox72*	This study
bBD113	*amyE::PspoIIQ-cfp(Bs) (cat), spoIIIE::lox72, spoIIQ::erm, ycgO::PspoIIQ-spoIIQ Y28A (kan)*	This study
bBD114	*amyE::PspoIIQ-cfp(Bs) (cat), spoIIQ::tet, ycgO::PspoIIQ-spoIIQ Y28A (kan)*	This study
bBD116	*amyE::PspoIIQ-cfp(Bs) (cat), spoIIIE::lox72, spoIIQ::erm, ycgO::PspoIIIE-spoIIIE*D584A(phleo)*	This study
bBD117	*amyE::PspoIIQ-cfp(Bs) (cat), spoIIEΩpKM282a(spoIIE-yfp(spec)) spoIIQ::tet, ycgO::PspoIIQ-spoIIQ Y28A (kan),*	This study
bBD132	*amyE::PspoIIQ-cfp(Bs) (cat), spoIIIAH::spec, spoIIIE:: lox72, spoIIQ::erm, ycgO::PspoIIQ-spoIIQ Y28A (kan)*	This study
bBD133	*amyE::PspoIIQ-cfp(Bs) (cat), spoIIIAH::spec, spoIIIE::kan*	This study
bBD137	*amyE::PspoIIQ-cfp(Bs) (cat), spoIIIE:: lox72, spoIIQ::erm, , ycgO::PspoIIIE-spoIIIE*D584A(phleo), yhdG:: PspoIIQ-spoIIQ Y28A (spec)*	This study
bBD144	*amyE::PspoIIQ-cfp(Bs) (cat), spoIIIAH::erm, spoIID::spec spoIIIE::neo, spoIIP::tet*	This study

bBD149	*amyE::PspoIIQ-cfp(Bs) (cat), gerM::erm, spoIIIE::kan*	
bBD150	*amyE::PspoIIQ-cfp(Bs) (cat), spoIID::spec, spoIIIAH::erm, spoIIIE:: lox72, spoIIP::tet spoIIQ::phleo, ycgO::PspoIIQ-spoIIQ Y28A (kan)*	This study
bBD158	*amyE::PspoIIQ-cfp(Bs) (cat), gpsB::kan*	This study
bBD164	*amyE::PspoIIQ-cfp(Bs) (cat), gpsB::kan, spoIIIE::phleo*	This study
Plasmids		
pBD012	*yhdG::PspoIIQ-spoIIQ Y28A (spec)*	This study

Plasmid Construction

pBD012 [*yhdG::PspoIIQ-spoIIQ Y28A*] was generated by a two-way ligation of a *Eco*RI-*Bam*HI of a gene block (IDT) containing the relevant DNA segment of SpoIIQ into pBB278 cut with *Eco*RI-*Bam*HI. pBB278 is an ectopic integration vector for double cross-over insertions into the non-essential *yhdG* locus (B. Burton and DZR, unpublished).
